# Enabling Highly Efficient and Stable Perovskite Photovoltaics via A Multidentate Molecular Anchor Additive

**DOI:** 10.1007/s40820-026-02098-8

**Published:** 2026-02-11

**Authors:** Liangding Zheng, Tai Wu, Lei Yang, Yong Hua

**Affiliations:** 1 Institute of International Rivers and Eco-Security, Yunnan University, Kunming, 650091 People’s Republic of China; 2 The Australian Centre for Advanced Photovoltaics, School of Photovoltaic and Renewable Energy Engineering, University of New South Wales, Sydney, NSW 2052 Australia; 3 Yunnan Key Laboratory for Micro/Nano Materials & Technology, School of Materials and Energy, Yunnan University, Kunming, 650091 People’s Republic of China

**Keywords:** Perovskite solar cell, Additive, Charge carrier, Dynamics

## Abstract

Suppressing formamidinium (FA) loss and perovskite phase degradation is very crucial for achieving highly efficient and long-term stable perovskite solar cells (PSCs). Herein, we designed and synthesized a novel multifunctional additive (ZL1) to stabilize *α*-FAPbI_3_ perovskite phase through synergistic multisite interactions: **i** its F atoms form F···H–N hydrogen bonds with FA^+^, (ii) its phenyl rings participate in cation–π interactions with FA^+^, (iii) the C=O and S groups coordinate Pb^2+^ through Lewis acid–base interactions, and (iv) the NH groups engage I^−^ anions through N–H···I hydrogen bonding. Consequently, ZL1 molecule can effectively suppress FA loss and optimizes perovskite crystallization kinetics, yielding high-quality and stable *α*-FAPbI_3_ perovskite films with enlarged grain sizes and reduced defect density. Meanwhile, ZL1 treatment promotes exciton dissociation, facilitates hole extraction from the perovskite layer into the hole transport layer, and reduces charge carrier recombination in device. The ZL1-modified device achieves a power conversion efficiency of 26.13%, significantly outperforming the control device (24.20%). A similar improvement is observed in wide-bandgap PSCs, with efficiency increasing from 18.44% to 20.53% after ZL1 treatment. Notably, the unencapsulated ZL1-based devices exhibit exceptional operational stability under both illumination and thermal conditions.

**Supplementary Information:**

The online version contains supplementary material available at 10.1007/s40820-026-02098-8.

## Highlights

- A novel multifunctional additive (ZL1) enables formamidinium loss suppression and perovskite stabilization via synergistic multisite interactions.
- ZL1 demonstrates defect passivation, enhanced charge carrier extraction, and reduced charge recombination.
- The optimized devices exhibit a high champion efficiency of 26.13%, combined with robust photothermal stability.

**Supplementary Information:**

The online version contains supplementary material available at 10.1007/s40820-026-02098-8.

## Graphical abstract

**Figure:**
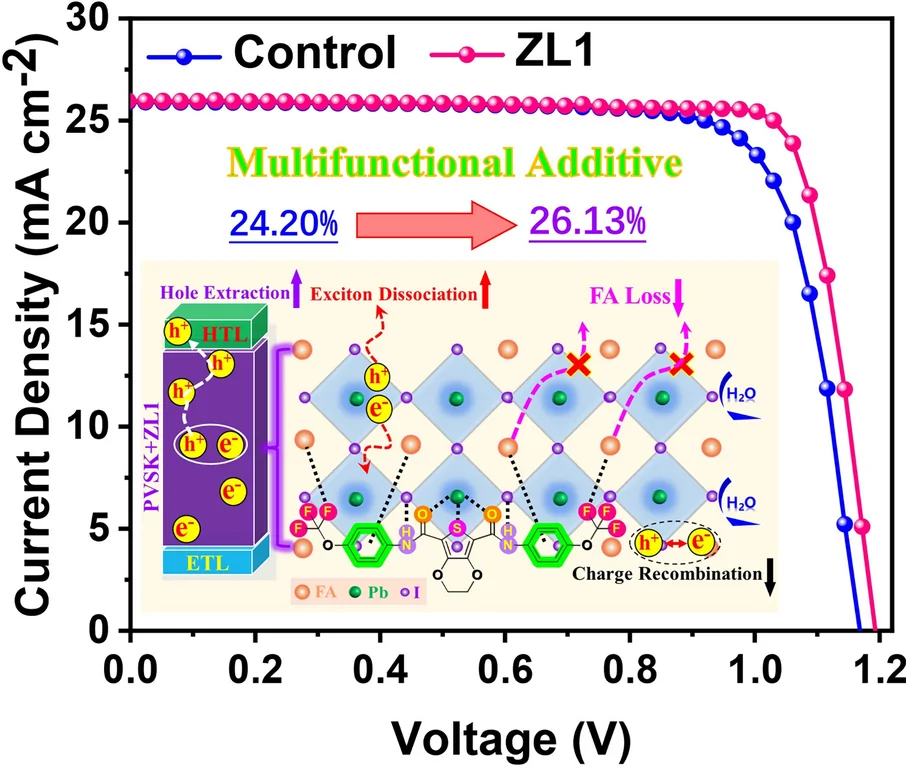

## Introduction

Perovskite solar cells (PSCs) have emerged as a groundbreaking technology in the field of photovoltaics, presenting a highly promising alternative to conventional silicon-based solar cells. Since 2009, PSCs have undergone extraordinary advancements with power conversion efficiencies (PCEs) soaring from the initial 3.8% to over 27% in just over a decade [1–4]. This remarkable progress is largely due to the distinctive optoelectronic properties of perovskite materials, including efficient light absorption ability, long charge carrier diffusion lengths, and high charge carrier mobility [5–8]. Among the various perovskite compositions, formamidinium (FA)-based perovskite (FAPbI_3_) has emerged as the most promising light-absorbing material for fabricating highly efficient single-junction and tandem PSCs owing to its near-ideal optical bandgap [9, 10]. Although FA-based PSCs have achieved remarkable PCEs at the laboratory scale, their commercialization is challenged by the inherent thermodynamic instability of the photoactive FAPbI_3_ phase under operational environments. A critical issue is the spontaneous phase transition of FAPbI_3_ from the photoactive cubic (*α*-phase, black) to a non-perovskite hexagonal (*δ*-phase, yellow) structure under ambient condition shortly, resulting in the serious decomposition of perovskite materials. It is well known that this phase transition of the black *α*-FAPbI_3_ is primarily driven by the organic component FA random orientation and evaporation from the perovskite framework due to the volatile nature of FA component [11, 12]. In particular, the loss of FAI often induces the formation of a high concentration of hydrogen iodide (HI). The HI is highly susceptible to oxidation into volatile I_2_ molecules, which subsequently leaves behind numerous iodine vacancies (V_I_^+^) in perovskite films. Meanwhile, the charge imbalance from FAI loss is compensated by the reduction of Pb^2+^ to form Pb^0^ defects. Consequently, widespread V_I_^+^ and Pb^0^ defects exist at grain boundaries and surface of perovskite films, which arises the non-radiative recombination and significantly hampers the charge carrier transport/extraction properties in devices; on account of this, the long-term operating stability deteriorates rapidly in these devices [13–15]. Therefore, immobilizing FA cations and suppressing their loss are very critical for enhancing FAPbI_3_ perovskite stability.

Some effective additives strategies have been explored to enhance the inherent stability of the black *α*-phase of FAPbI_3_. Yi et al. partially substituted FA^+^ with Cs^+^ ions, which stabilized the perovskite structure without significantly altering the bandgap, leading to improved device stability under ambient conditions and high temperature [11]. Yang et al. incorporated 2D phenylethylammonium lead iodide into *α*-FAPbI_3_ to form a 2D/3D heterostructure, which enhanced perovskite moisture resistance while suppressing FA migration [16]. Grätzel et al. demonstrated that 5-ammonium valeric acid iodide can stabilize *α*-FAPbI_3_ by forming hydrogen bonds with the 3D perovskite lattice, suppressing the *α*-to-*δ* phase transition. This results in highly crystalline films with micrometer-sized grains, improved charge carrier transport, and reduced non-radiative recombination [17]. A work by Kim et al. showed that employing pseudohalide anion formate as a stabilizer could maintain the *α*-FAPbI_3_ against environmental degradation and suppress anion vacancy defects at the grain boundaries and surface of perovskite films that significantly enhanced the device's stability and performance [18]. Nevertheless, current additives weakly interact with FA only through single-site bonding [15, 19]. Meanwhile, these strategies often neglect the crucial role of simultaneous coordination with Pb^2+^ and I^−^ ions in passivating these Pb_i_ and V_I_^+^ defects. Consequently, they remain inadequate for achieving effective and durable stabilization of *α*-phase FAPbI_3_ perovskite films.

**Fig. 1:**
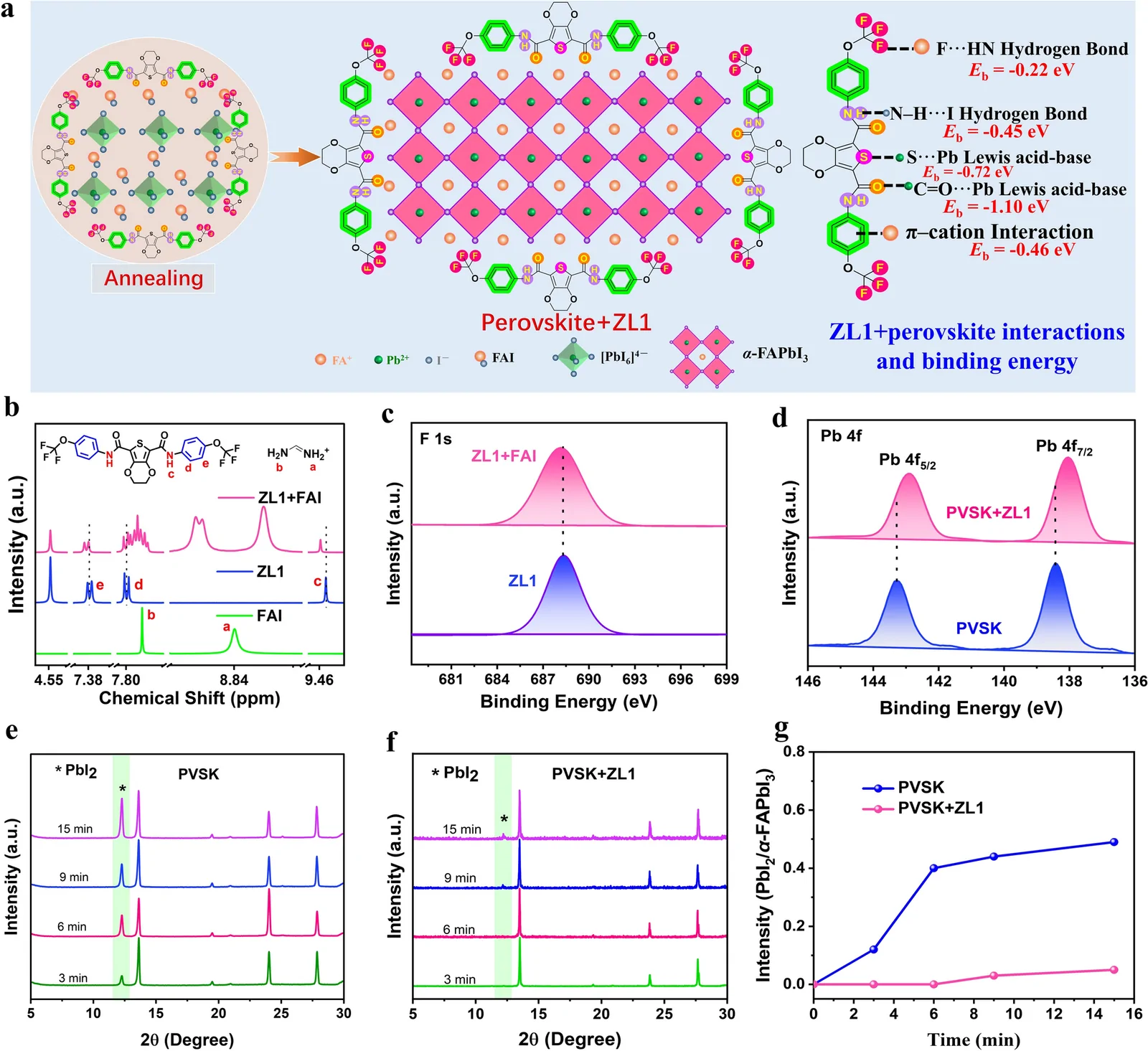
**a** Molecular structure design of ZL1 and schematic diagram of the interactions and binding energy between ZL1 and perovskite. **b** ^1^H NMR spectra of FAI, ZL1 and ZL1 mixed with FAI. **c** XPS spectra (F 1*s*) of ZL1 and ZL1 + FAI. **d** XPS spectra (Pb 4*f*) of ZL1 and perovskite (PVSK) + ZL1. XRD of **e** control and **f** ZL1-treated perovskite films under different annealing times. **g** Peak intensity ratio of PbI_2_/(perovskite (100) peak) of control and ZL1-based perovskite films under different annealing times

To summarize, developing novel multifunctional additives molecules with multiple active sites that can simultaneously interact with FA^+^, Pb^2+^, and I^−^ ions to suppress FA cations loss and achieve robust stabilization of *α*-FAPbI_3_ black phase has remained a critical challenge in perovskite photovoltaics. In this work, we designed and synthesized an organic small molecule (ZL1), featuring multiple active sites as a multifunctional additive to stabilize the black phase of *α-*FAPbI_3_ via FAI immobilization strategy. As illustrated in Fig. 1a, the F atoms and phenyl groups in ZL1 can interact with FA^+^ via F···H–N hydrogen bonding and π–cation interactions, respectively. Meanwhile, the C = O and S groups coordinate with Pb^2+^ ions through Lewis acid–base interactions, while the NH groups form hydrogen bonds with I^−^ anions. Consequently, perovskite films treated with ZL1 exhibit enhanced stabilization of the *α*-FAPbI_3_ black phase, yielding high-quality films with reduced defects. This optimization significantly improves devices’ efficiency and stability.

## Experimental Section

### Materials

Tin dioxide aqueous solution (SnO_2_, 15 wt%) was purchased from Alfa Aesar. Formamidinium lead triiodide (FAPbI_3_) black powder, FAI, PbBr_2_, DMAI, CsI, PI, and MACl were purchased from Greatcell solar. NiO_x_, C60, Me-2PACz, Spiro-OMeTAD, and octylammonium iodide were purchased from Advanced Election Technology. Lithiumbis-(trifluoromethylsulfonyl) imide (Li-TFSI, 99%), 4-tertbutyl-pyridine (tBP), N,N-dimethylformamide (DMF, 99.8%), BCP, chlorobenzene (CB, 99.8%), acetonitrile (99.8%), dimethyl sulfoxide (DMSO, ≥ 99.9%) and diethyl ether were purchased from Sigma-Aldrich. 2,3-Dihydrothieno[3,4-b][1,4]dioxine-5,7-dicarboxylic acid and 4-(trifluoromethoxy)aniline were purchased from Aladdin. All of the above materials were used without further purification treatment.

### Device Fabrication

#### Narrow-Bandgap n-i-p PSC Fabrication

The glass/ITO substrates were cleaned thoroughly by sequential ultrasonication for 20 min in a detergent solution, distilled water, alcohol, and acetone. Then, the substrates were dried with N_2_ and cleaned with UV ozone for 30 min before use. The SnO_2_ colloid solution was spin-coated onto the cleaned ITO substrates at 4000 rpm for 30 s, followed by annealing at 150 °C for 30 min. Perovskite films with varying compositions were fabricated using the spin-coating method, as detailed below. Subsequently, HTM solution (90 mg Spiro-OMeTAD, 37 μL tBP, 44 *μ*L Li-TFSI (260 mg Li-TFSI in 1 mL acetonitrile), and 9 μL of Co-TFSI salt (375 mg mL^−1^ in acetonitrile), dissolved in 1 mL CB) is spin-coated onto the interface layer with a spin-coating process 4000 rmp for 30 s. Finally, 70 nm Au electrode was thermally evaporated onto the hole transport layer under high vacuum to obtain complete devices.

*FAPbI*_*3*_* perovskite films*: 1.4 M FAPbI_3_ and 35 mol % MACl in a mixture of DMF and DMSO (8:1). For ZL1-doped perovskite, 2 mg ZL1 was added into 1 mL perovskite solution. The filtered perovskite solution was spread over the as-prepared SnO_2_ substrate at 6500 rpm for 50 s with a ramping duration of 0.1 s. During the spin-coating process, 200 μL of diethyl ether as an anti-solvent was dripped after spinning for 10 s, followed by immediate annealing on a hot plate at 150 °C for 15 min. 4 mg mL^−1^ of octylammonium iodide dissolved in IPA was spin-coated on the top of the perovskite films at 4000 rpm for 30 s and annealed at 100 °C for 5 min.

*FA*_*0.94*_*Cs*_*0.06*_*PbI*_*3*_* perovskite films*: The FA_0.94_Cs_0.06_PbI_3_ perovskite precursor solution was prepared by mixing FAI, CsI and PbI_2_ with 800 µL DMF and 200 µL DMSO according to the stoichiometric ratio. Then, 2 mol% CsCl was introduced to increase the crystallinity. For ZL1-doped perovskite, 2 mg ZL1 was added into 1 mL perovskite solution. The filtered perovskite solution was spread over the as-prepared SnO_2_ substrate at 5000 rpm for 35 s. During the spin-coating process, 120 μL of ethyl acetate as an anti-solvent was dripped after spinning for 20 s, followed by immediate annealing on a hot plate at 150 °C for 15 min. For the interfacial passivation layer, piperazine dihydriodide in IPA (0.5 mg mL^−1^) was spin-coated on top of perovskite films (5000 rpm, 30 s) and annealed at 100 °C for 5 min.

#### Wide-Bandgap p-i-n PSC Fabrication

The glass/ITO substrates were cleaned thoroughly by sequential ultrasonication for 20 min in a detergent solution, distilled water, alcohol, and acetone. Then, the substrates were dried with N_2_ and cleaned with UV ozone for 30 min before use. NiOx particle water solution (15 mg mL^−1^) was prepared by spin-coating the filtered solution onto ITO substrates at 3000 rpm for 30 s and annealing the films at 120 °C for 15 min. Subsequently, the Me-2PACz in ethanol solution (1 mg mL^−1^) was spin-coated at 3000 rpm for 30 s and annealed at 100 °C for 10 min. 1.5 M FA_0.78_Cs_0.15_DMA_0.07_Pb(I_0.7_Br_0.3_)_3_ precursor solution was prepared by dissolving PbI_2_, PbBr_2_, FAI, DMAI, and CsI in a solvent mixture (V_DMF_: V_DMSO_ = 4:1) and stirring overnight at 60 °C. Perovskite layer was prepared by spin-coating the solution at 1000 rpm for 2 s and then 5000 rpm for 30 s. 200 μL CB was added as an anti-solvent at 20 s. The wet films were annealed at 100 °C for 60 min. For ZL1-doped perovskite, 2 mg ZL1 was added into 1 mL perovskite solution. Then, 3 mg mL^−1^ of F-PEAI IPA solution was spin-coated onto the perovskite surface at 4000 rpm for 30 s and annealed at 100 °C for 5 min. Finally, it is transferred to a high-vacuum chamber (6 × 10^−4^ Pa), and the C60 (30 nm, 0.1 Å s^−1^), BCP (8 nm, 0.1 Å s^−1^), and Ag (120 nm, 0.2 Å s^−1^) are thermally evaporated.

## Results and Discussion

### Perovskite Film Characterizations

ZL1 molecule was synthesized via a straightforward two-step reaction sequence, as outlined in Scheme S1. The chemical structure of ZL1 was unambiguously confirmed by nuclear magnetic resonance (^1^H NMR and ^19^F NMR) spectroscopy (Figs. S1 and S2). To gain insight into the interaction between ZL1 and perovskite films, ^1^H NMR spectroscopy measurements were initially taken, as shown in Fig. 1b. Notably, pronounced chemical shift perturbations in the ^1^H NMR spectra were observed upon mixing FAI with ZL1, suggesting the potential intermolecular coordination interactions between the two components. The ^1^H NMR spectrum shows two distinct sharp peaks at 8.84 and 7.88 ppm, which are unambiguously assigned to the characteristic hydrogen resonances of NH_2_^+^ (labeled **a**) and NH_2_ (labeled **b**) in FAI, respectively. Upon addition of ZL1, a pronounced downfield shift is observed for these characteristic signals: The peak initially at 8.84 ppm shifts to 8.99 ppm, while the signal at 7.88 ppm splits into two well-resolved proton resonances at 8.64 and 8.67 ppm. These changes indicate an altered chemical environment of the corresponding hydrogen atoms. Upon mixing ZL1 with FAI, the proton resonance of the -NH group (labeled **c**) shifts slightly downfield from 9.49 to 9.46 ppm, which strongly indicates the formation of an N–H···I hydrogen bond between ZL1 and FAI. To further verify this N–H···I interaction, the ^1^H NMR spectra of ZL1 and ZL1-PbI_2_ were compared (Fig. S3). After mixing with PbI_2_, the -NH signal in ZL1 shifts from 9.49 to 9.45 ppm with relative to the pristine ZL1. These results confirm the formation of a N–H···I hydrogen bond between the N–H group in ZL1 and the I atom in FAI. Additionally, the chemical shifts observed at approximately 7.25 and 7.82 ppm corresponding to the aromatic protons (labeled **d** and **e**) of the phenyl groups in ZL1. Upon mixing with FAI, obvious shifts in these aromatic proton signals are observed in the ZL1 + FAI sample, demonstrating the formation of the cation–π interaction between FA^+^ cations and phenyl groups [20]. Meanwhile, we also performed ^19^F NMR spectra on ZL1 without and with FAI. As shown in Fig. S4, the addition of FAI in ZL1 sample induces an obvious ^19^F signal shift from -57.01 to -56.99 ppm, suggesting that the F groups in ZL1 could interact with FAI.

To further understand the interaction between ZL1 and perovskite, X-ray photoelectron spectroscopy (XPS) analysis was conducted. As shown in Fig. 1b, the F 1*s* peak in ZL1 + FAI sample shifts to lower binding energy of 687.97 eV from 688.35 eV for ZL1 sample, which confirms the formation of hydrogen bonding of F···H–N between ZL1 and FAI [21, 22]. Subsequently, we further probed the F···H–N hydrogen bonding by Raman spectrum. As shown in Fig. S5, it is found that the C-F vibration of ZL1 shifts from 1257.06 to 1259.92 cm^−1^ after mixing with FAI, which confirms the hydrogen bonding interaction. Additionally, the XPS N 1*s* peak (400.03 eV) of ZL1 sample moves toward a lower binding energy of 399.88 eV after mixing with FAI (Fig. S6), suggesting the potential formation of -NH···I hydrogen bond between the -NH groups in ZL1 and I^−^ ions in FAI [23]. Furthermore, as displayed in Fig. 1c, the two Pb 4*f*_7/2_ and Pb 4*f*_5/2_ peaks in perovskite films treated with ZL1 show a noticeable shift to lower binding energies with relative to that of the pristine perovskite films. Raman results (Fig. S7) show that an obvious shift of the C = O stretching vibration is seen in ZL1 + PbI_2_ sample with relative to the ZL1-based one. Further analysis of the sulfur Raman spectra (Fig. S8) reveals a shift in the S-related peak of ZL1 from 749.10 to 748.07 cm^−1^ after mixing with PbI_2_. This shift is attributed to a coordination interaction between the sulfur (S) group in ZL1 and the Pb^2+^ ions. These findings totally demonstrate that the C = O and S functional groups in ZL1 form the coordination bonds with Pb ions in the perovskite films via C = O···Pb and S···Pb interactions [24]. Taken together, these results demonstrate that ZL1 with multifunctional groups can result in the formation of the hydrogen bonding and coordination interactions with these FA^+^, Pb^2+^, and I^−^ ions in perovskite, which are expected to immobilize these ions and reduce the vacancy defects. To further verify the coordination interactions, the density functional theory (DFT) calculations was carried out. As shown in Fig. S9a, the ZL1 molecule exhibits a linear structure. Its interaction with the perovskite lattice was further investigated through DFT energy relaxation in a 1 × 1 × 1 supercell with a single ZL1 molecule anchored on an FAPbI_3_ (001) slab. The intermolecular spacing distance of ZL1 (21.220 Å) is more than three times the lattice constant of the optimized perovskite along the *a/b/c* axes (6.3613 Å, Fig. S9b), indicating effective interaction between ZL1 and the lattice through anchoring. After geometry optimization, ZL1 adopts a parallel orientation relative to the perovskite lattice (Fig. S9c–e). The calculated binding energy (*E*_b_) between ZL1 and the perovskite is − 2.10 eV, demonstrating strong cooperative interaction. The electron density plots and side-view structural images (Fig. S10, top and bottom, respectively) reveal that ZL1 anchors to the perovskite via a set of five distinct interactions (S…Pb and C = O···Pb Lewis acid–base interaction, NH···I hydrogen bonding, cation–π interaction, and F–FA interaction). The corresponding *E*_b_ values are –0.72, –1.10, –0.45, –0.46, and –0.211 eV, respectively. Thus, these DFT results further confirm the existence of these interactions between ZL1 and perovskite.

Organic component FAI in perovskite has been previously demonstrated as a key factor of contributing to PSCs instability owing to its photosensitivity, tendency to oxidize into I_2_ and I_3_^−^ species, and inherent volatility [25, 26]. We conducted ultraviolet–visible (UV–Vis) absorption spectroscopy to investigate the potential stabilizing effect of ZL1 on FAI. As shown in Fig. S11 (inset), the FAI solution turns into light yellow coloration after 30 min of light exposure, whereas the FAI + ZL1 solution maintains its transparency in color. Moreover, it is found that two additional absorption peaks at 290 nm (assigned to I_2_) and 360 nm (assigned to I_3_^−^) are observed in FAI solution under light in contrast to the individual ZL1 or FAI + ZL1 solutions. These results clearly demonstrate that the incorporation of ZL1 can effectively suppress the FAI oxidative and photodecomposition (I_2_ and I_3_^−^formation), which will be help for significantly improving FAI and perovskite stabilities under light illumination operation. The crystallographic changes of perovskite under different annealing durations were further explored by X-ray diffraction (XRD) spectra. As exhibited in Fig. 1e, f, a strong diffraction peak at around 13.94° is seen in both samples, which corresponds to the (100) crystal plane of perovskite [27]. The diffraction peak at 12.65° corresponds to PbI_2_ phase. In the pristine perovskite films (Fig. 1e), the XRD spectra reveal a gradual increase in the intensity of the PbI_2_ diffraction peaks with prolonged annealing time, suggesting progressive decomposition of the perovskite phase into PbI_2_. In contrast, when ZL1 is incorporated into the perovskite, the annealed films exhibit only minor PbI_2_ formation, as evidenced by the significantly weaker PbI_2_ diffraction signals in Fig. 1f. To further quantify the phase evolution, we compared the intensities ratio of the PbI_2_ and perovskite (100) diffraction peaks extracted from the XRD spectra. As exhibited in Fig. 1g, the intensity ratio of the control films continuously increases with annealing time, while the ZL1-treated films show remarkable stability with only a minor variation in the intensity ratio. The results unambiguously demonstrate that ZL1 treatment effectively suppresses the undesirable perovskite phase decomposition and PbI_2_ formation by inhibiting thermal-induced FA evaporation during annealing, which originates from ZL1’s interaction with FAI.

**Fig. 2:**
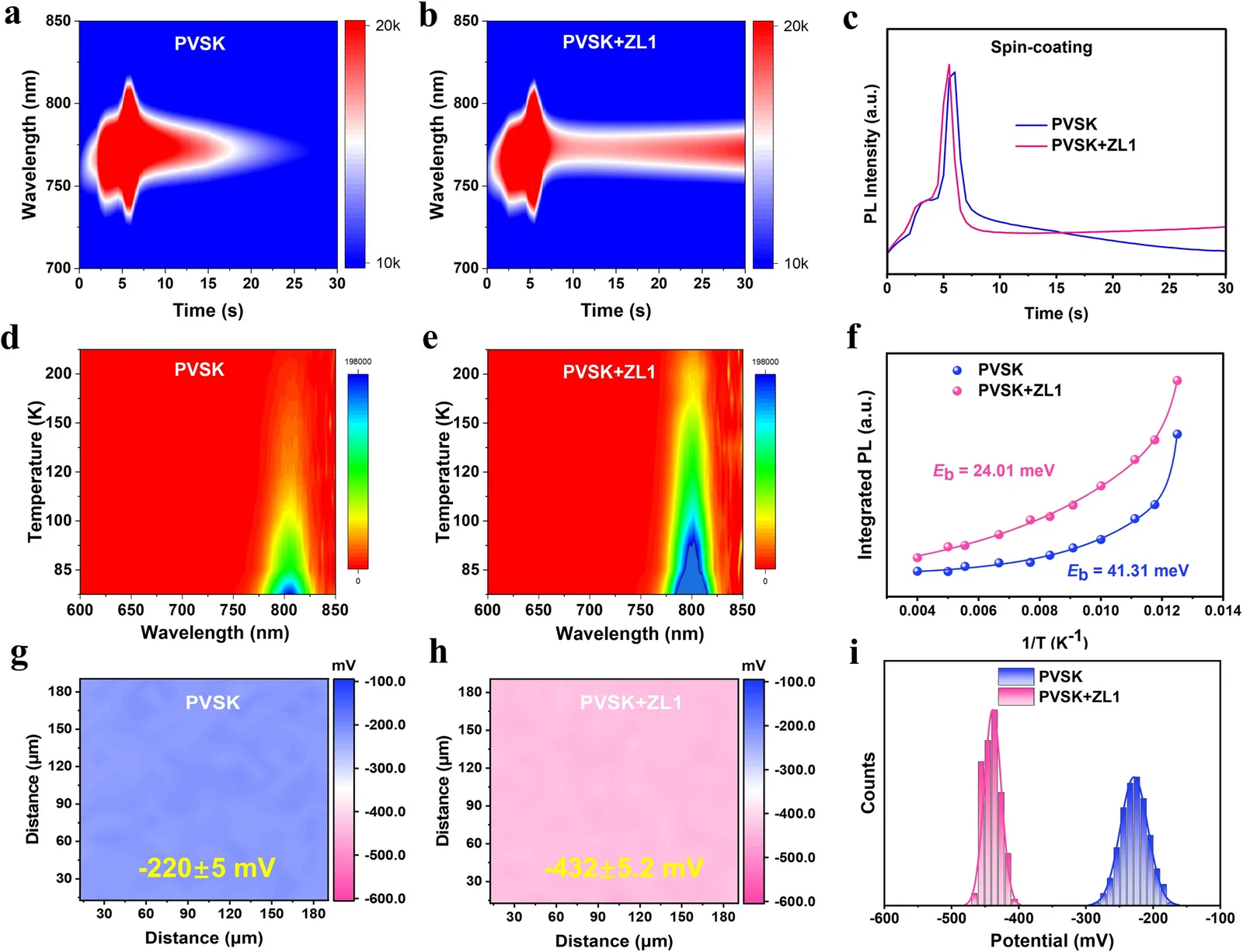
In situ PL 2D spectra of **a** perovskite and **b** perovskite + ZL1 films during annealing. **c** In situ PL spectra of perovskite films during annealing. Temperature-dependent PL pseudocolor maps of **d** perovskite and **e** perovskite + ZL1 films. **f** Exciton binding energies derived from temperature-dependent PL emission spectra. Surface potential images of **g** control and **h** ZL1-treated perovskites from KPFM. **i** Statistical potential distributions of perovskite films surfaces

To investigate the influence of ZL1 on perovskite crystallization, we performed in situ photoluminescence (PL) of perovskite films during the spin-coating process. As shown in Fig. 2a, the control films exhibit strong PL emission at the initial 15 s, suggesting the reaction between PbI_2_ and organic salts FAI. Subsequently, the PL intensity decreases rapidly in the control sample under annealing. In contrast (Fig. 2b), ZL1-treated films maintain persistent PL emission throughout spin-coating process. Notably, the PL intensity rapidly rises in ZL1-based perovskite films that occur 1 s earlier than that in the control films, as exhibited in Fig. 2c. This observation clearly indicates that ZL1 can facilitate high-quality perovskite formation with the reduced non-radiative recombination [28]. We further employed scanning electron microscopy (SEM) and atomic force microscopy (AFM) to investigate the morphologies of perovskite films under varying annealing periods. As illustrated in SEM images (Fig. S12), both the control and ZL1-based perovskite films display fully formed perovskite grains after annealing for 3 min. When the annealing time is extended to 15 min, the control films exhibit relatively inferior and uneven morphologies containing some grain boundaries (GBs) and unreacted PbI_2_ owing to the FA evaporation during high-temperature annealing. However, the ZL1-treated perovskite films achieve high-quality morphologies with larger grain size and less GBs. Importantly, no PbI_2_ is detected in these films as the thermal annealing time is prolonged. These results demonstrate that the addition of ZL1 can effectively suppress FA cations loss and perovskite decomposition under thermal annealing. Moreover, as shown in AFM images (Fig. S13), we could observe that perovskite films with ZL1 show more uniform surface morphologies with larger grain size with relative to the control films, which is well consistent with the observed SEM results. Thus, high-quality ZL1-treated perovskite films is beneficial for reducing perovskite defects and facilitating the charge carrier transfer in device.

Then, we employed the space charge limited current (SCLC) technique to investigate the defect density of perovskite films. As shown in Fig. S14a–d, the ZL1-treated perovskite films show much lower trapping–filling limit voltage (*V*_TFL_) and defect density (*N*_trap_) than that of the control films, which suggests that ZL1 can effectively passivate these defects within films, promoting charge carrier transfer and simultaneously inhibiting charge recombination in device [29]. Additionally, temperature-dependent PL spectra were used to investigate the exciton binding energy (*E*_b_) of perovskite films. Figure 2d, e exhibits the pseudocolor PL spectra of perovskite films across temperatures from 70 to 230 K. It is found that the PL intensity obviously enhances for the ZL1-treated perovskite films with relative to the pristine films across various temperatures, further confirming reduced defect density and charge carrier recombination after ZL1 treatment. Figure 2f compares the *E*_b_ values of both samples, as determined by the Arrhenius equation [30, 31]:

1 $$I(T)=\frac{{I}_{0}}{1+A{e}^{-{E}_{b/({k}_{B}T)}}}$$

where *I*(*T*) and *I*_0_ represent the PL integrated intensity at *T* and 0 K, respectively, and *k*_B_ represents Boltzmann's constant. ZL1-treated perovskite films demonstrate a significantly reduced *E*_b_ of 24.01 meV compared to the control films (41.31 meV). This reduction in *E*_b_ can result in faster exciton dissociation, consequently promoting charge carrier transfer in device. Furthermore, Kelvin probe force microscopy (KPFM) was utilized to study the surface electronic properties of perovskite films. Figure 2g–h displays the KPFM maps on the top surface of perovskite films, and their corresponding statistical potential histograms are exhibited in Fig. 2i. Prior to KPFM measurements, the system was first calibrated with a gold reference. As shown in Fig. S15, 400-point measurements on the surfaces of all three samples yield straight-line plots, indicating good reproducibility. We found that ZL1-treated perovskite films exhibit narrower potential distribution compared with the pristine films, suggesting a smoother perovskite surface. Moreover, the reference perovskite films show an average potential value of approximately − 220 mV, which is significantly shifted down to -430 mV after ZL1 treatment, demonstrating more efficient charge carrier generation in ZL1-treated perovskite layer [32, 33], which will be beneficial to transport the charge carriers to hole transport layer (HTL) from perovskite layer, thus leading to less charge recombination at the perovskite/HTL interface and enhanced PSCs performance.

### Device Performance and Analysis

**Fig. 3:**
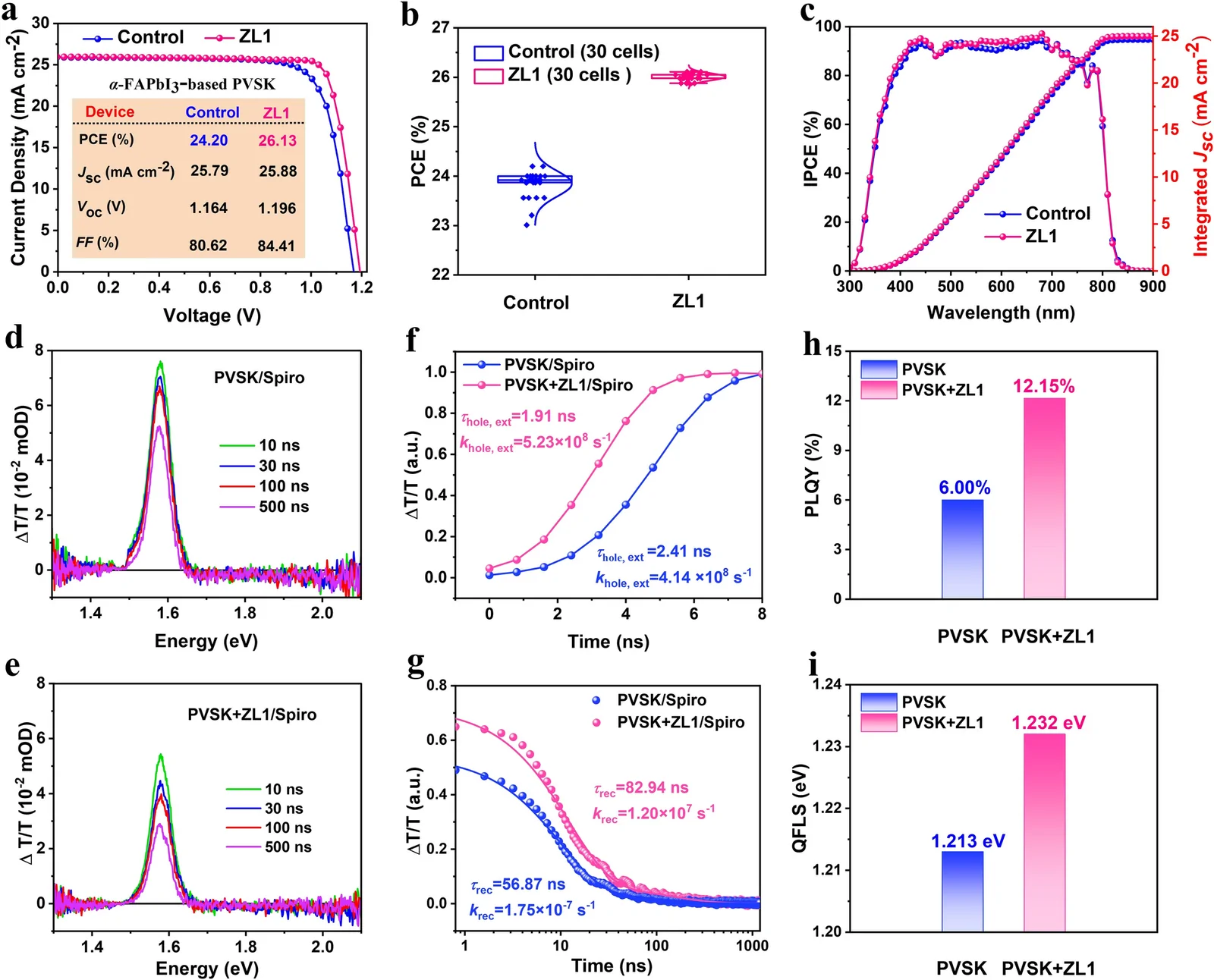
**a** *J–V* curves of FAPbI_3_-based devices with and without ZL1. **b** PCE statistical data of 30 FAPbI_3_-based devices. **c** EQE spectrum and integrated *J*_SC_ of the FAPbI_3_-based devices. The ns-TA spectra of **d** perovskite (PVSK)/Spiro-OMeTAD (Spiro) and **e** PVSK + ZL1/Spiro interfaces. **f** Hole extraction kinetics curve of PVSK/Spiro without and with ZL1. **g** Charge recombination kinetics curve of PVSK/Spiro without and with ZL1. **h** PLQY of perovskite films without and with ZL1. **i** QFLS values of perovskite films without and with ZL1

To investigate the influence of ZL1 onto PSCs photovoltaic performances, we fabricated PSCs based on a configuration of ITO/SnO_2_/PVSK/Spiro-OMeTAD/Au. Figure 3a presents the photocurrent density–voltage (*J–V*) characteristics of devices without and with ZL1 recorded under the simulated AM 1.5G illumination. Optimization of the devices was first performed by varying ZL1 concentration. The *J–V* characteristics (Fig. S16) reveal that the highest PCE was obtained at the ZL1 concentration of 2.0 mg mL^−1^. As shown in steady-state photoluminescence (PL) spectra (Fig. S17), the strongest PL peak intensity at 2 mg mL^−1^ indicates the lowest trap-assisted non-radiative recombination. AFM measurements further reveal that ZL1 doping enlarges perovskite grains and reduces surface roughness (Fig. S18). The optimal concentration of 2 mg mL^−1^ yields the largest grains and the lowest root-mean-square (RMS) roughness of perovskite films. The PCE of the control device is 24.20% along with a current density (*J*_SC_) of 25.79 mA cm^−2^, an open-circuit voltage (*V*_OC_) of 1.164 V, and a fill factor (FF) of 80.62%. The PCE is significantly enhanced to 26.13% (*J*_SC_ = 25.88 mA cm^−2^, *V*_OC_ = 1.196 V, and FF = 84.41%) for the ZL1-treated device. As shown in Fig. 3b, the PCE statistical analysis reveals that the ZL1-based devices demonstrate significantly enhanced performance, achieving a higher average PCE of 26.05% compared to 23.92% for control devices, along with higher *V*_OC_ and FF values (Figs. S19 and S20) in ZL1-treated devices. In addition, the ZL1-treated device shows smaller hysteresis than that of the control device, as exhibited in Fig. S21 and Table S1. Figure S22 presents the stabilized power outputs of devices measured at the maximum power point. It is revealed that the ZL1-modified devices maintain a higher efficiency output of 26.05% over a duration of 500 s, whereas the control devices exhibit a lower stabilized efficiency of 23.78%. These results demonstrate that the incorporation of the ZL1 molecule can effectively enhance photovoltaic performance of device. The integrated *J*_SC_ values derived from the external quantum efficiency (EQE) measurements (Fig. 3c) is 25.12 and 25.04 mA cm^−2^ for the control and ZL1-treated devices, respectively, closely matching the measured *J*_SC_. ZL1 molecule was incorporated into 1 cm^2^ device. The resulting ZL1-based device achieves a higher PCE of 24.24% than that of the control device (22.91%), as shown in Fig. S23. We then evaluated the impact of ZL1 on the performance of 1.76 eV bandgap PSCs. As shown in *J–V* curves (Fig. S24), the control device shows a PCE of 18.44%, with a *V*_OC_ of 1.212 V, a *J*_SC_ of 19.66 mA cm^−2^, and a FF of 77.39%. In contrast, the ZL1-treated PSCs achieves an enhanced PCE of 20.53%, along with a *V*_OC_ of 1.279 V, a *J*_SC_ of 19.83 mA cm^−2^, and an FF of 80.93%, in which the *J*_SC_ values show good agreement with the integrated currents from the EQE spectrum (Fig. S25). Figure S26 presents the *J–V* characteristics of FA_0.94_Cs_0.06_PbI_3_ PSCs. The pristine device demonstrates a PCE of 23.80%, whereas the device modified with the ZL1 additive shows an improved PCE of 25.38%. The demonstration of improved photovoltaic performances in devices verifies the effectiveness and universality of ZL1 as a perovskite additive.

To understand the enhanced performance of device after ZL1 treatment, steady photoluminescence (PL) and time-resolved photoluminescence (TRPL) spectra were performed. As shown in Fig. S17, the perovskite films treated with ZL1 exhibit significantly enhanced PL intensity compared to the pristine films. This improvement can be attributed to the suppression of non-radiative recombination in ZL1-treated perovskite films, resulting from both the improved film quality and reduced defect density [32]. Figure S27 shows the time-resolved photoluminescence (TRPL) spectroscopy of perovskite films without and with ZL1 under different laser intensities ranging from 0.06 to 0.23 μJ cm^−2^. It is clearly found that the perovskite films treated with ZL1 demonstrate extended charge carrier lifetimes with relative to their untreated counterparts, which further confirms lower non-radiative recombination rate, consequently achieving higher *V*_OC_ in ZL1-based device. To elucidate the charge carrier transfer dynamics, including hole extraction and charge recombination process, nanosecond transient absorption (ns-TA) spectroscopy was employed to investigate the PVSK/Spiro-OMeTAD films without and with ZL1. The corresponding ns-TA spectra recorded over delay times from 10 to 500 ns are presented in Fig. 3d, e. The ns-TA spectra exhibit a strong characteristic ground-state bleach (GSB) for both samples, corresponding to the band-filling effect [34]. The ΔT/T signals of the PVSK + ZL1/Spiro films exhibit a slight attenuation compared with the PVSK/Spiro-based films at equivalent delay times. This observation implies a reduced hole population at the PVSK + ZL1/Spiro interface [35], which can be attributed to more efficient hole extraction from the ZL1-treated perovskite layer into the Spiro-OMeTAD-based hole transport layer. Figure 3f presents the TA decay kinetics curves of these samples, which is analyzed to evaluate the hole extraction dynamics. The obtained hole extraction time constants (*τ*_hole, ext_) are determined to be 2.41 and 1.91 ns for of the of the PVSK/Spiro and PVSK + ZL1/Spiro films, respectively. Thus, the corresponding hole extraction rate (*k*_hole, ext_ = 1/*τ*_hole, ext_ = 5.23 × 10^8^ s^−1^) for PVSK + ZL1/ Spiro films is higher than that of the PVSK/Spiro sample (*k*_hole, ext_ = 4.14 × 10^8^ s^−1^), demonstrating a significant enhancement in hole extraction efficiency with ZL1 incorporation. Furthermore, the charge carrier recombination dynamics, as shown in Fig. 3g, reveals that the PVSK + ZL1/Spiro sample exhibits a longer charge carrier lifetime (*τ*_rec_ = 82.94 ns) and lower recombination rate (*k*_rec_ = 1.20 × 10^7^ s^−1^) for the PVSK + ZL1/Spiro sample compared with the control PVSK/Spiro sample (*τ*_rec_ = 56.87 ns and *k*_rec_ = 1.75 × 10^7^ s^−1^). These ns-TA results clearly demonstrate that the introduction of ZL1 effectively facilitates hole extraction from the perovskite layer to the Spiro-OMeTAD-based hole transport layer, and consequently suppressing charge carrier recombination, which contributes to the enhanced photovoltaic performance observed in the ZL1-treated device.

**Fig. 4:**
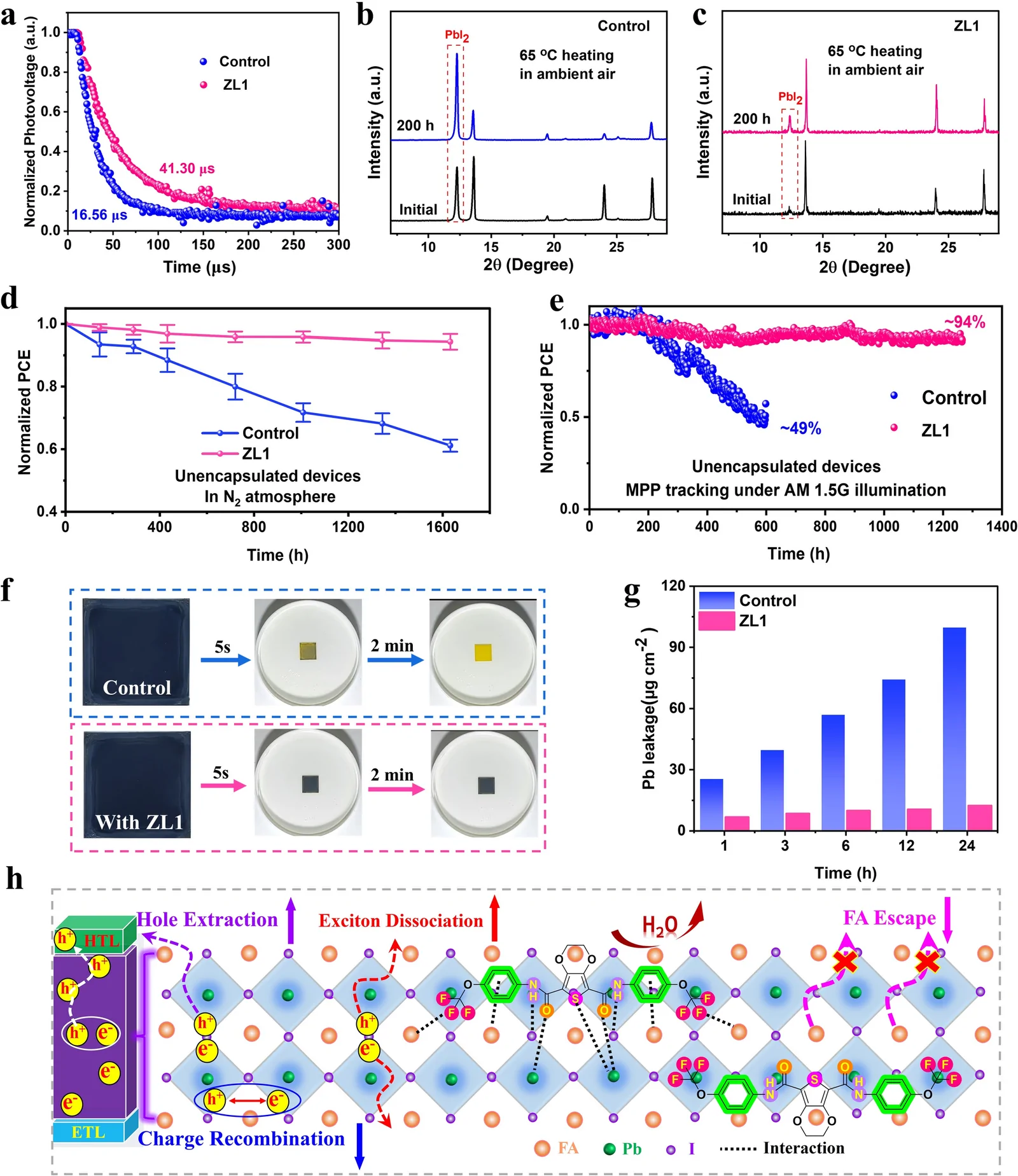
**a** TPV curves of devices. XRD spectra of **b** the control perovskite films and **c** ZL1-treated perovskite films under 65 °C heating in ambient air (RH: 35% ~ 45%). **d** Long-term stability of unencapsulated devices stored in N_2_ glovebox. **e** Long-term stability of unencapsulated devices under MPP tracking under AM 1.5G illumination. **f** Photographs of the control and ZL1-modified perovskite films in water immersion measurements. **g** Evolution of the Pb^2+^ concentration of perovskite films during deionized water immersion. **h** Schematic diagram of the charge carrier dynamics

To gain deeper insight into the improved performance of PSCs after ZL1 treatment, we performed photoluminescence quantum yield (PLQY) measurements and estimated the quasi-Fermi level splitting (QFLS). As shown in Fig. 3h, the ZL1-treated perovskite films exhibit a higher PLQY value of 12.15% than that of the control films (6.00%). Furthermore, the QFLS value increases from 1.213 eV (control) to 1.232 eV after ZL1 treatment, as exhibited in Fig. 3i. These results clearly demonstrate that ZL1 can effectively suppress the charge carrier recombination, thereby contributing to the enhancement of *V*_OC_ in the targeted device [36]. The findings are further supported by electrochemical impedance spectroscopy (EIS) measurements (Fig. S28), where the ZL1-based PSCs displays significantly higher charge recombination resistance than that of the control device, which suggests effective suppression of non-radiative recombination, thereby improving the *V*_OC_ and FF values [37]. To further investigate the charge recombination kinetics, we measured the light intensity dependence of* J*_SC_ (Fig. S29). Clearly, the exponential factor (*α*) of the ZL1-treated device is increased to 0.996 from 0.968 for the control device, representing the suppressed non-radiative recombination in ZL1-modified device [38]. Additionally, dark current–voltage analysis (Fig. S30) shows the reduce leakage current in ZL1-treated device compared to the control device, indicating the lower charge recombination rate in ZL1-treated device [39]. We further analyzed the charge carrier dynamics process of device by transient photovoltage (TPV) measurements. As illustrated in Fig. 4a, the ZL1-treated device shows a remarkable 2.5-fold enhancement in charge carrier lifetime of 41.30 μs with relative to the control device of 16.56 μs, further unambiguously demonstrating the suppressed charge recombination in the ZL1-modified device [39]. Based on the comprehensive characterization results, we conclude that the ZL1 treatment effectively suppresses the charge carrier recombination, collectively contributing to the enhanced *V*_OC_ and FF values in ZL1-based device, which ultimately results in the improved PSC performances.

PSCs devices instability often originate from perovskite degradation, particularly its decomposition into PbI_2_. The decomposition behavior of perovskite films under 65 °C heating in ambient air (humidity: 35% ~ 45%) was assessed by XRD and SEM measurements. As illustrated in XRD results (Fig. 4b, c), the control perovskite films exhibit a marked increase in PbI_2_ diffraction peak at 12.65°, indicating severe perovskite degradation. For comparison, the ZL1-treated perovskite films show only a minimal PbI_2_ diffraction peak in XRD patterns after 200 h, demonstrating the remarkable stability of perovskite films. Additionally, SEM observations (Fig. S31) reveal that the pristine perovskite films exhibit a rough surface morphology with numerous pinholes and voids. In contrast, the perovskite films treated with ZL1 possess relatively smooth and pinhole-free surfaces with reduced roughness. Under continuous 1-sun illumination in ambient air for 200 h, ZL1-treated perovskite films underwent only a minor increase in PbI_2_ peak intensity in XRD spectra (Fig. S32); however, the control films suffer a dramatic rise. Consistent with this, SEM images (Fig. S33) show that the control perovskite films underwent clear decomposition with fragmented surfaces under 1-sun illumination in ambient air, whereas the ZL1-modified films show a smooth morphology with large perovskite grains. As shown in thermogravimetric analysis (Fig. S34), the onset of decomposition shifts from approximately 188 °C for pristine FAI to 255 °C for the FAI–ZL1 composite, which could contribute to enhance the thermal stability of FAI. These results demonstrate that ZL1 incorporation can effectively suppress perovskite decomposition, thereby significantly enhancing the operational photothermal stability of devices.

We subsequently conducted a systematic investigation of the operational stabilities of these unencapsulated devices. As illustrated in Fig. 4d, the control PSCs exhibit significant degradation, retaining merely 63% of its initial PCE after 1600 h under N_2_ atmosphere. In contrast, the ZL1-modified devices demonstrate exceptional stability, maintaining 97% of the original efficiency over the identical duration. Figure S35 shows the thermal stability of devices after ambient storage at a temperature of 65 °C. The ZL1-modified device exhibits outstanding thermal stability, retaining 96% of the initial performance after 720 h, while the control device shows substantial degradation (65% PCE retention). Furthermore, we quantitatively evaluated the photostability of the devices through maximum power point (MPP) tracking under continuous 1-sun illumination (AM 1.5G, 100 mW cm^−2^), as shown in Fig. 4e. Remarkably, the ZL1-treated PSCs demonstrate exceptional operational stability, retaining 94% of the initial PCE after 1300 h of continuous illumination. In contrast, the control devices exhibit rapid degradation, maintaining only 49% of initial performance after just 600 h. The MPP photostability results show good agreement with the actual *J–V* characteristics measured before and after the MPP measurements (Fig. S36). These findings collectively validate the efficacy of the ZL1 modification, significantly improving the operational stabilities of devices.

The toxicity of lead and its potential leakage from perovskites pose a major barrier to its widespread commercialization. First, perovskite films without and with ZL1 were immersed in deionized water in order to evaluate their moisture stability. Detailed procedures of the immersion test are provided in Movies S1 and S2. As shown in photographs of the control and ZL1-modified perovskite films in water immersion (Fig. 4f), the control films began decomposing into PbI_2_ within 5 s of water immersion, which becomes more pronounced after 2 min. In contrast, the ZL1-treated perovskite films maintain the black perovskite phase under the same working conditions. Subsequently, the intensity of perovskite films immersed in water was further characterized by employing UV–Vis spectroscopy (Fig. S37). Clearly, the reference perovskite film exhibits a rapid decrease in absorption intensity after only 2 min of water exposure, accompanied by the emergence of PbI_2_, indicating severe decomposition of the perovskite phase. However, the ZL1-based perovskite films maintain high absorption intensity, demonstrating no detectable PbI_2_ formation and significantly improved resistance to water. In order to quantify the Pb^2+^ ions leaching into deionized water, the Pb^2+^ ion concentrations of water solution containing perovskite films were measured after immersion periods of 1, 3, 6, 12, and 24 h using inductively coupled plasma atomic emission spectrometry. We calculated the lead ion concentration for each time point by applying the standard calibration curve equation (Fig. S38) ( S28). Figure 4g displays the variation in Pb^2+^ concentration level over time. We observed that the lead leakage from the control perovskite films increase significantly from 1 to 24 h, whereas the ZL1-treated films exhibit much lower concentration of Pb^2+^ ions in water under the same conditions. Notably, after 24 h of water immersion, the Pb^2+^ leakage concentration from the ZL1-modified perovskite films is much lower than that of the control sample, decreasing sharply from 100 to 12 µg cm^−2^. Upon immersion in deionized water containing Ca^2+^ and Mg^2+^ ions (Fig. S39), lead leakage from the control perovskite films increases substantially over 5 h, whereas ZL1‑treated films exhibit much lower Pb^2+^ concentrations, confirming that the inhibitory effect of ZL1 on lead leakage remains effective despite the presence of divalent ions. These results indicate that incorporating ZL1 into perovskite films can effectively inhibit perovskite phase degradation and suppress the release of Pb^2+^ ions owing to its strong coordination interaction with FA^+^, Pb^2+^, and I^−^ ions, which further contributes to the enhanced stability of PSCs. As illustrated in Fig. 4h, our findings lead us to conclude that the ZL1 incorporation can effectively suppress FA escape and perovskite phase decomposition by concurrently interacting with FA^+^, Pb^2+^, and I^−^ species, thereby strongly stabilizing perovskite phase and enhancing device stability. Moreover, the resulting ZL1-treated perovskite films exhibit superior morphology with enlarged grains and fewer defects, which synergistically facilitates exciton dissociation and hole extraction from the perovskite layer into the hole transport layer and reduces charge carrier recombination, consequently resulting in the enhanced device performance.

## Conclusions

In summary, we have designed and synthesized a multifunctional organic small-molecule additive (ZL1) for perovskite solar cell application. This compound features multiple coordination sites that enable simultaneous interactions with FA^+^ cations, Pb^2+^ ions, and I^−^ anions in FAPbI_3_-based perovskite, effectively stabilizing the perovskite crystal structure. We found that the F atoms and phenyl groups in ZL1 interact with FA^+^ via F···H–N hydrogen bonding and cation–π interactions, respectively. Meanwhile, the C=O and S groups coordinate with Pb^2+^ ions through Lewis acid–base interactions, while the NH groups form hydrogen bonds with I⁻ anions. Consequently, perovskite films treated with ZL1 exhibit the enhanced stabilization of the *α*-FAPbI_3_ black phase. Moreover, the incorporation of ZL1 can facilitate the reaction between PbI_2_ and FAI, which accelerates the perovskite nucleation and crystallization, consequently yielding high-quality perovskite films with enlarged perovskite grains and reduced defect density. Additionally, dynamics studies found that ZL1 treatment promotes efficient charge carrier separation and facilitates hole extraction from the perovskite layer into the hole transport layer, efficiently reducing charge carrier recombination in device. As a result, the PSCs treated with ZL1 achieves a champion PCE of 26.13%, significantly higher than the 24.20% of the control device. Furthermore, when incorporated into wide-bandgap PSCs, ZL1 boosts the efficiency from 18.44% to 20.53%, validating its broad effectiveness and universality. Notably, the unencapsulated ZL1-based device exhibits exceptional operational stability under continuous 1-sun illumination condition. ZL1-based PSCs maintain 94% of its initial efficiency for 1300 h, while the control PSCs retain only 49% of its origin efficiency.

## Supplementary Information

Below is the link to the electronic supplementary material.

Supplementary file1 (DOCX 9357 KB)

Supplementary file2 (MP4 2229 KB)

Supplementary file3 (MP4 3290 KB)
