# Supplementary material for: Enabling Highly Efficient and Stable Perovskite Photovoltaics via A Multidentate Molecular Anchor Additive
Source: Nanomicro Lett. 2026 Feb 11;18:250. doi: 10.1007/s40820-026-02098-8 (PMC12891301; doi:10.1007/s40820-026-02098-8)
Supplement: Supplementary file 1 — Supplementary file1 (DOCX 9357 KB) [file 40820_2026_2098_MOESM1_ESM.docx]

Supporting Information for

**Enabling Highly Efficient and Stable Perovskite Photovoltaics via A Multidentate Molecular Anchor Additive**

Liangding Zheng^1,‡^, Tai Wu^2,‡^, Lei Yang^1^, Yong Hua^1,3,^*

^1^ Institute of International Rivers and Eco-security, Yunnan University, Kunming 650091, P. R. China

^2^ The Australian Centre for Advanced Photovoltaics, School of Photovoltaic and Renewable Energy Engineering, University of New South Wales, Sydney, New South Wales 2052, Australia

^3^ Yunnan Key Laboratory for Micro/Nano Materials & Technology, School of Materials and Energy, Yunnan University, Kunming 650091, P. R. China

^‡^ Liangding Zheng and Tai Wu contributed equally to this work.

*Corresponding author. E-mail: [huayong@ynu.edu.cn](mailto:huayong@ynu.edu.cn) (Yong Hua)

**S1 Experimental Section**

**S1.1 Synthesis**

**
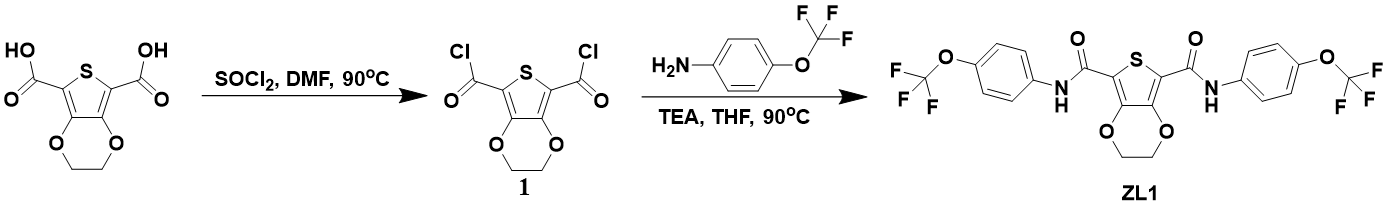
**

**Scheme S1** The synthesis route of ZL1

**ZL1**. A mixture of 2,3-dihydrothieno[3,4-b][1,4]dioxine-5,7-dicarboxylic acid (2.30 g, 10.00 mmol) and thionyl chloride (25 mL) was refluxed, in the presence of catalytic amount of DMF, at 90℃for 12 h. The resultant light-green solution was cooled to room temperature and the excess thionyl chloride was removed under reduced pressure. The residue was washed with hexane (3×15 mL) and dried by vacuum to yield the intermediate 1, Yield: 2.37 g (89%). This compound 1 was used for preparation of ZL1 without further purification. 4-(trifluoromethoxy)aniline (1.32 g, 7.48 mmol), 1 (1.0 g, 3.74 mmol), and triethylamine (4.0 mL; 28.7 mmol) in a solution mixture of anhydrous THF (50 mL). The reaction mixture is stirred overnight at 90°C under nitrogen. Evaporate the solvent under vacuum. The white solid residue is washed with acetone, water, then filtered and dried overnight under vacuum. ZL1 was purified by column chromatography on silica gel. Yield: 1.88 g (92%). ^1^H NMR (600 MHz, DMSO-*d*, δ): 9.49 (s, 2H), 7.81 (d, J = 8.46 Hz, 4H), 7.40 (d, J = 8.42 Hz, 4H), 4.56 (s, 4H). ^1^F NMR (600 MHz, DMSO-*d*, δ): 57.12.

**S1.2 Characterization of perovskite films and devices**

NMR experiments were conducted on a Bruker Avance III spectrometer. XPS spectra were obtained by X-ray Photoelectron Spectroscopy (K-Alpha+, 1486.6 eV). XRD patterns of the perovskite films were obtained by the rotating target X-ray polycrystal diffractometer (RIGAKUTTRIII-18KW). Scanning electron microscopy (SEM) images were collected using a scanning electron microscope (FEI NOVA NANO-450). Atomic force microscope (AFM) images were obtained by tapping mode by Cypher ES. The UV-vis absorption spectra were obtained by UV-vis spectrometer (CSPM5500). SSPL and TRPL spectra were obtained by FLS1000 at 475nm excitation wavelength. Photoluminescence quantum yield (PLQY) was performed via using FLS1000 at 475nm excitation wavelength. The *J-V* curves and steady-state power outputs of the devices were obtained at 100 mW/cm^2^ (AM 1.5G) using a 2400 source meter and a Newport Solar Simulator (Model 91160) and calibrated with a standard silicon-based solar cell prior to use. Incident photon-current conversion efficiency (IPCE) spectra were obtained by means of a series of setup: a monochromator (Spectra Products CM110), a Xenon light source (Spectral Products ASB-XE-175), and a potentiostat (Lab Jack U6 DAQ board). Electrochemical experiments (e.g., TPC and EIS) were performed with ZAHNER (PP211). ns-TAS was measured by LP980. First, a 355nm laser is generated by a triple frequency Nd:YAG laser (Spectra-Physics Quanta-Ray ProSeries, 10 Hz repetition rate, 10 ns pulse length), and then a 355nm laser (5 μJ/cm^2^) is pumped through a pump optical parametric oscillation (OPO) to generate a 475nm pump Pu laser. The white light is provided by the XBO 450 W xenon arc lamp (OSRAM). The camera, detector and oscilloscope are iStar CCD camera (Andor Technology) and LP980-K photomultiplier tube (PMT) detector and Tektronix MDO 3022 200 MHz 2.5 GS/s respectively. In this work, the space charge limiting current (SCLC) measurements with a device structure (ITO /SnO_2_/Perovskite/PCBM/Ag) were performed to analyze the trap density (*N*_trap_) of perovskite films with and without ZL1 treatment. *N*_trap_ can be calculated using the following equation:

$$N_{trap=}(2\varepsilon\varepsilon_{0}V_{TFL)}/eL^{2}$$

where *ε*_0_ is the vacuum permittivity of 8.8543×10^-12^ CV^-1^m^-1^, *ε* is the dielectric constant of perovskite for which 46.9 is adopted, e is the elementary charge, and L is the thickness of the perovskite films (L = 620 nm, measured by a profilometer).

**S1.3 Lead Ion Adsorption Experiments**

Inductively coupled plasma atomic emission spectrometry (ICP-AES) was employed to investigate the capability of ZL1 molecules to inhibit lead ion leakage. First, blank control experiments were performed on both the substrates (glass/ITO/tape) and the deionized water. No detectable lead signal was observed in either case. Subsequently, a series of lead acetate aqueous solutions (1, 2, 3, 4, 5, 10, 15, 20, and 25 μg/mL) were prepared to establish a standard calibration curve. The data exhibited a strong linear correlation between emission intensity and lead ion concentration upon linear fitting. Afterwards, different ITO/perovskite films were separately immersed in a glass dish (10.18 cm^-2^) containing 10 mL deionized water, and samples were collected at gradient time points for emission intensity detection. The lead ion concentration at each time point was calculated according to the standard calibration curve equation. For the lead leakage experiments involving different interfering ions (Mg^2+^ and Ca^2+^) with the concentration of both at 10 μg/mL were selected as the interfering ion sources.

**S2 Supplementary Figures and Tables**

**Fig. S1** ^1^H NMR (DMSO-*d*) spectrum of ZL1

**Fig. S2** ^19^F NMR (DMSO-d6) spectrum of ZL1

**Fig. S3** ^1^H NMR spectrum of ZL1 and ZL1+PbI_2_

**Fig. S4** ^19^F NMR spectrum of ZL1 and ZL1+FAI

**Fig. S5** Raman spectra (C-F) of ZL1 and ZL1+FAI

**Fig. S6** XPS spectra (N 1s) of ZL1 and ZL1+FAI

**Fig. S7** Raman spectra (C=O) of ZL1 and ZL1+PbI_2_

**Fig. S8** Raman spectra (S) of ZL1 and ZL1+PbI_2_


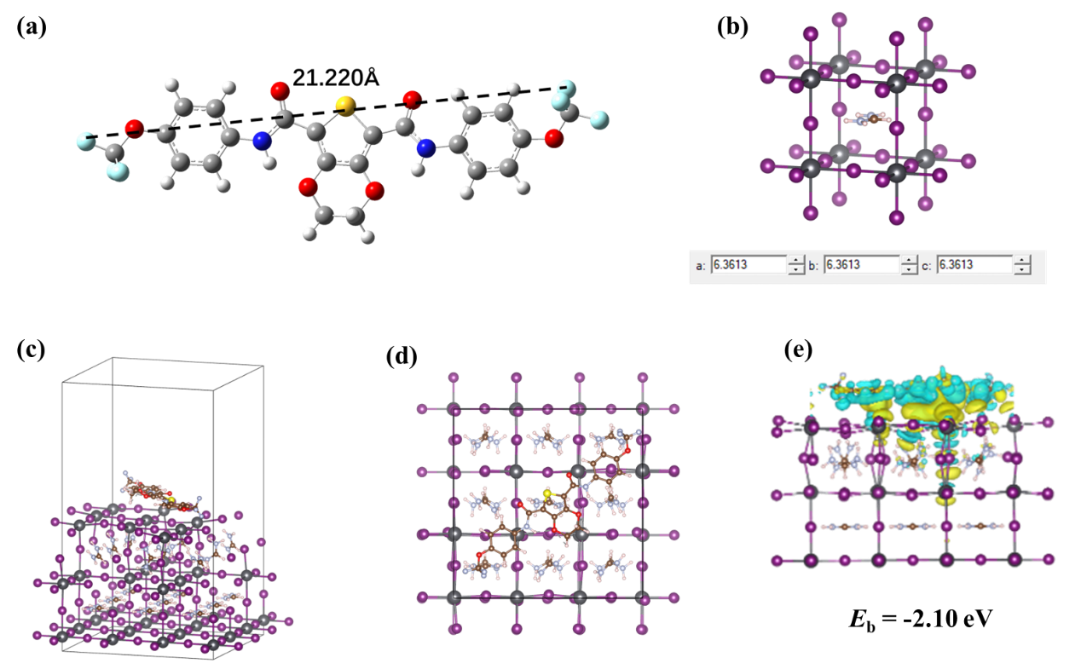


**Fig. S9** (**a**) The optimized ground-state molecular configuration of ZL1 and its intermolecular spacing distance. (**b**) FAPbI_3_ perovskite lattice parameters. Side-view (**c**) and top-view (**d**) images of the calculated structures of ZL1 interacting with perovskite. (**e**) Calculated *E*_b_ of ZL1 molecule with perovskite in parallel configuration


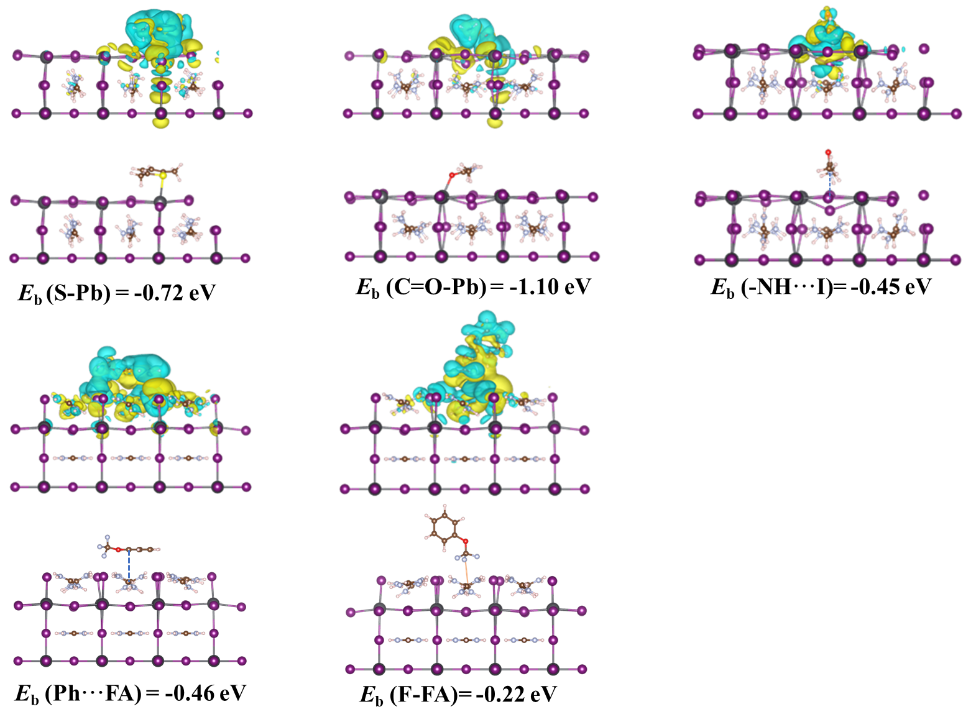


**Fig. S10** Electron-density distribution plots (top) and side-view structural images (bottom) of the ZL1 molecule interacting with the perovskite, illustrating the corresponding binding energies

**Fig. S11** UV–Vis absorption spectra of FAI and FAI+ZL1 in isopropyl alcohol solutions under light aging


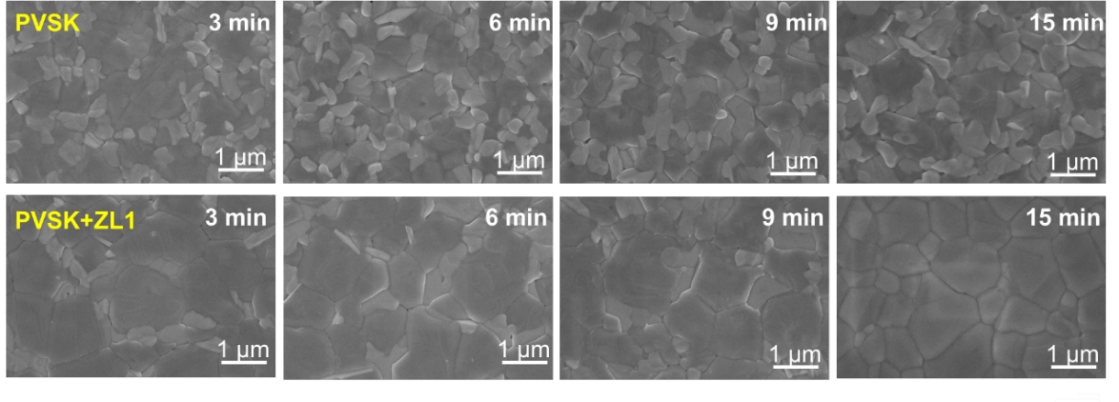


**Fig. S12** SEM images of perovskite films without and with ZL1 under different annealing time


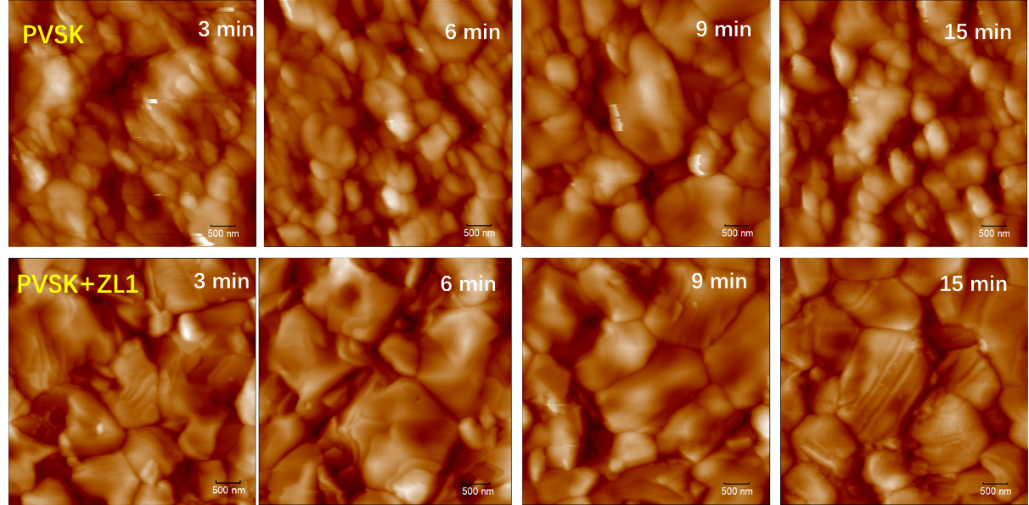


**Fig. S13** AFM images of perovskite films without and with ZL1 under different annealing time


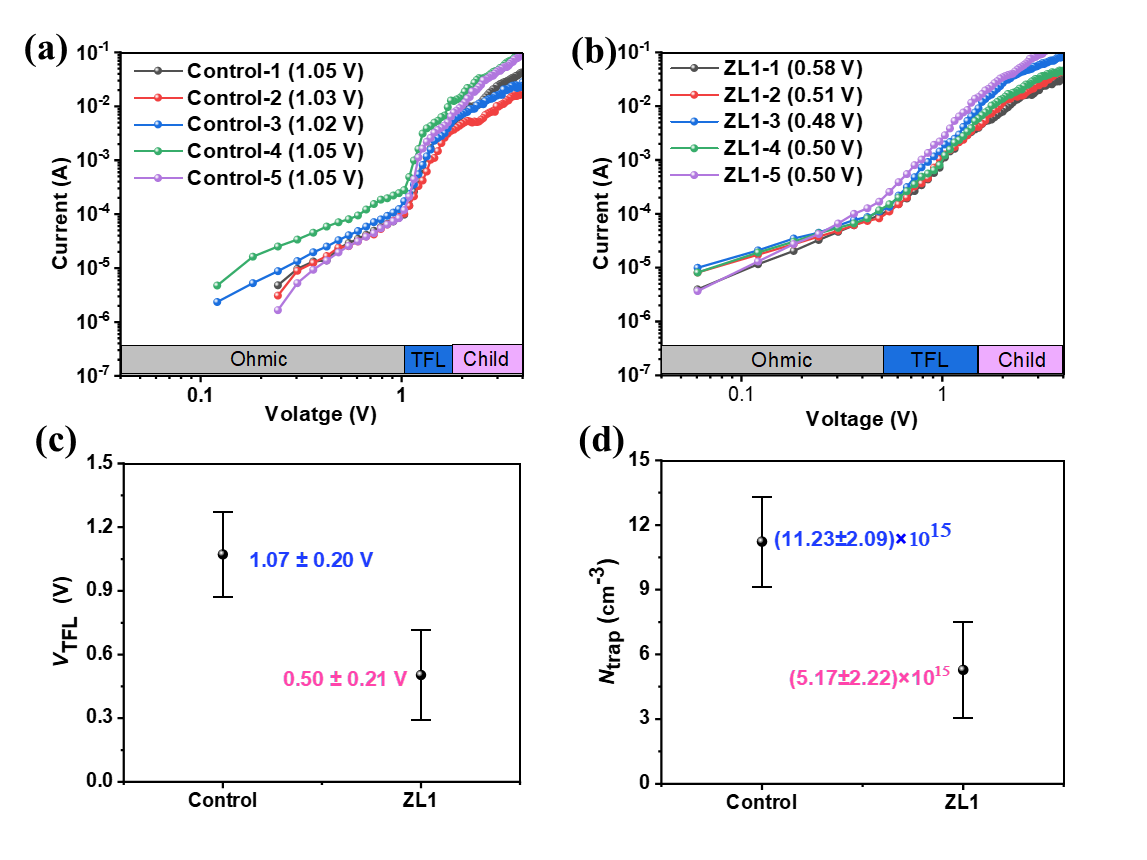


**Fig. S14** J-V curves from SCLC measurements of the electron-only devices without (**a**) and with ZL1 (**b**); (**c**) *V*_TFL_ error bars; (**d**) *N*_trap_ error bars

**Fig. S15** Surface photovoltage under different point

**Fig. S16** The *J-V* curves of devices with different ZL1 concentration

**Fig. S17** The stable PL of perovskite films with different ZL1 concentration


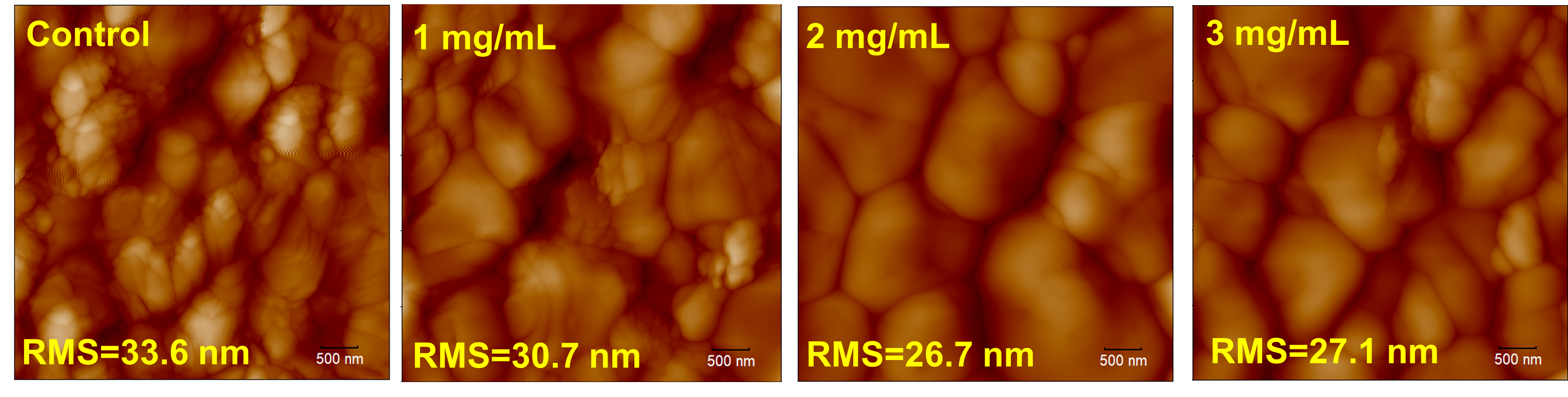


**Fig. S18** AFM images of perovskite films with different ZL1 concentration

**Fig. S19** *V*_OC_ statistical date of devices

**Fig. S20** *V*_OC_ statistical date of devices

**Fig. S21** *J–V* curves of the devices under reverse and forward scanning

**Fig. S22** Stabilized power output of devices

**Fig. S23** *J–V* curves of 1 cm^-2^ devices

**Fig. S24** *J-V* curve of the 1.76 eV devices

**Fig. S25** EQE spectrum and integrated current density of the1.76 eV devices

**Fig. S26** *J–V* curves of FACs-based PSCs


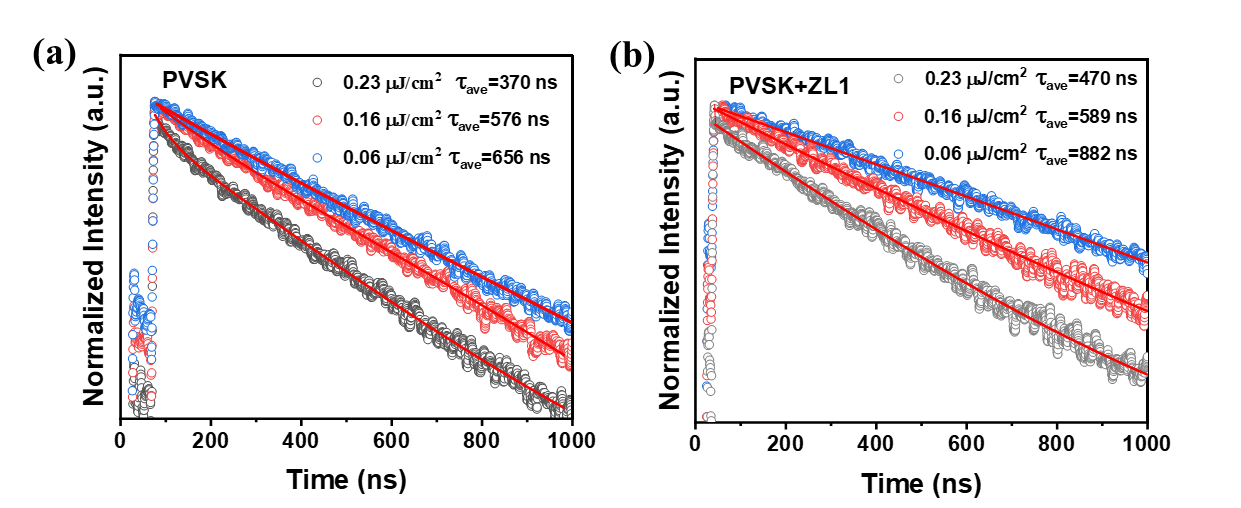


**Fig. S27** TRPL spectra of pristine perovskite (**a**) and perovskite+ZL1 (**b**) films under different laser intensities

**Fig. S28** EIS measurements under dark conditions

**Fig. S29** Light intensity-dependent *J*_SC_ plots of the control and ZL1-treated devices

**Fig. S30** Dark J-V curves of devices without and with ZL1


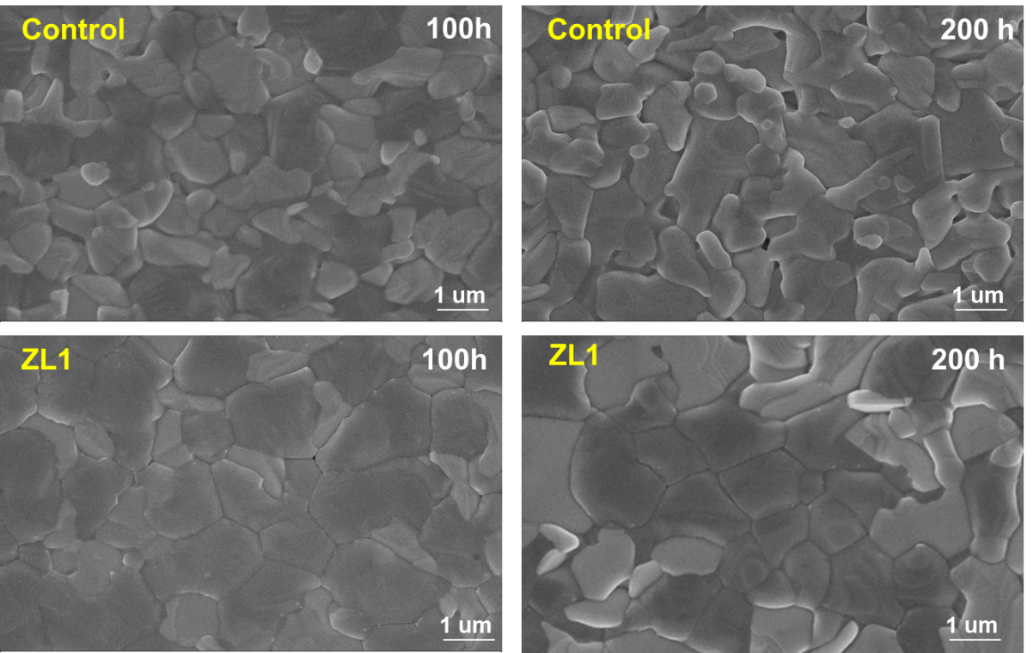


**Fig. S31** Surface SEM images of perovskite films under 65°C heating in air

**Fig. S32** XRD spectra of perovskite films without and with ZL1 under 1-sun illumination in ambient air for 200 hours


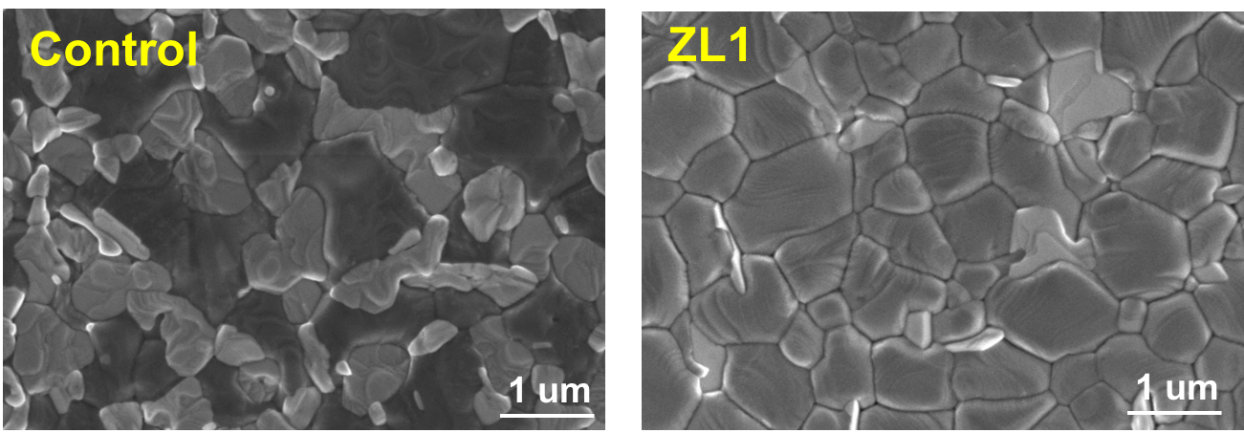


**Fig. S33** SEM images of perovskite films without and with ZL1 under 1-sun illumination in ambient air for 200 hours

**Fig. S34** TGA curve of FAI and FAI+ZL1

**Fig. S35** The thermal stabilities of the control and ZL1-treated devices

**Fig. S36** The actual *J-V* curves of the control and ZL1-treated devices before and after the MPP measurement

**Fig. S37** UV-vis spectra of control and ZL1-treated perovskite films before and after deionized water immersion stability testing


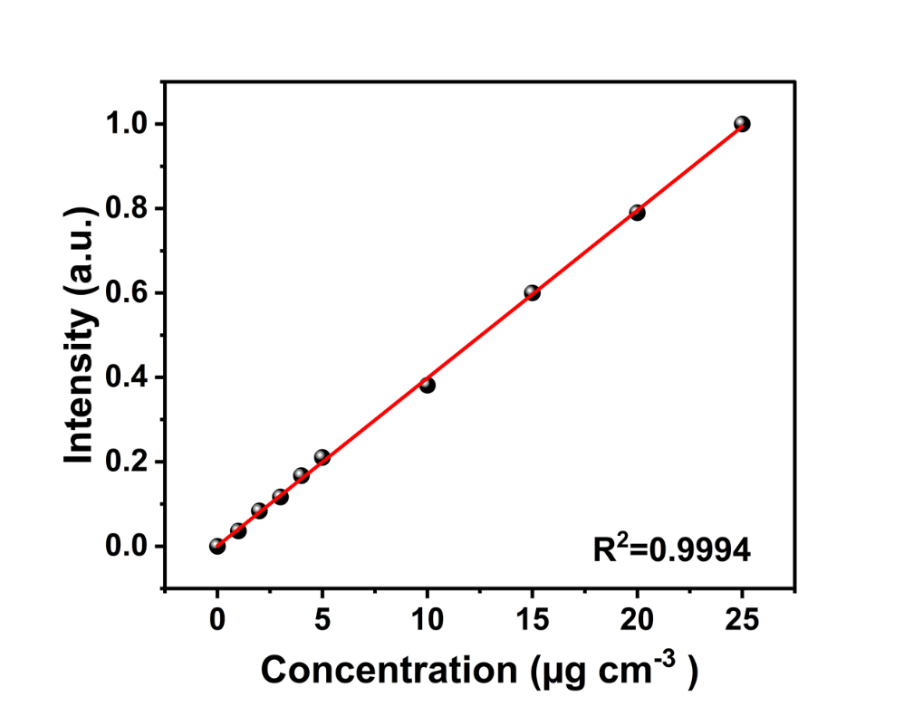


**Fig. S38** Pb^2+^ linear calibration curve

**Fig. S39** Evolution of the Pb^2+^ concentration of perovskite films in deionized water with Ca^2+^ and Mg^2+^

**Table S1** *J–V* curves of the devices under reverse and forward scanning

| Device | *J*_SC_ (mA cm^-2^) | *V*_OC_ (V) | FF (%) | PCE (%) |
| --- | --- | --- | --- | --- |
| Control (Forward) | 25.74 | 1.141 | 78.63 | 23.09 |
| Control (Reverse) | 25.79 | 1.164 | 80.62 | 24.20 |
| ZL1 (Forward) | 25.87 | 1.189 | 84.17 | 25.89 |
| ZL1 (Reverse) | 25.88 | 1.196 | 84.41 | 26.13 |
